# Digestion and Absorption of Milk Phospholipids in Newborns and Adults

**DOI:** 10.3389/fnut.2021.724006

**Published:** 2021-08-18

**Authors:** Åke Nilsson, Rui-Dong Duan, Lena Ohlsson

**Affiliations:** ^1^Division of Medicine, Gastroenterology, Department of Clinical Sciences Lund, Lund University, Lund, Sweden; ^2^Gastroenterology and Nutrition Laboratory, Division of Medicine, Department of Clinical Science, Lund University, Lund, Sweden; ^3^Division of Medicine, Experimental Vascular Medicine, Department of Clinical Science, Lund University, Lund, Sweden

**Keywords:** ceramidase, milk, phosphatidylcholine, phosphatidylethanolamine, phospholipase, sphingomyelin, sphingomyelinase

## Abstract

Milk polar lipids provide choline, ethanolamine, and polyunsaturated fatty acids, which are needed for the growth and plasticity of the tissues in a suckling child. They may also inhibit cholesterol absorption by interacting with cholesterol during micelle formation. They may also have beneficial luminal, mucosal, and metabolic effects in both the neonate and the adult. The milk fat globule membrane contains large proportions of sphingomyelin (SM), phosphatidylcholine (PC), and phosphatidylethanolamine (PE), and some phosphatidylserine (PS), phosphatidylinositol (PI), and glycosphingolipids. Large-scale technical procedures are available for the enrichment of milk fat globule membrane (MFGM) in milk replacement formulations and food additives. Pancreatic phospholipase A2 (PLA2) and mucosal phospholipase B digest glycero-phospholipids in the adult. In the neonate, where these enzymes may be poorly expressed, pancreatic lipase-related protein 2 probably has a more important role. Mucosal alkaline SM-ase and ceramidase catalyze the digestion of SM in both the neonate and the adult. In the mucosa, the sphingosine is converted into sphingosine-1-phosphate, which is both an intermediate in the conversion to palmitic acid and a signaling molecule. This reaction sequence also generates ethanolamine. Here, we summarize the pathways by which digestion and absorption may be linked to the biological effects of milk polar lipids. In addition to the inhibition of cholesterol absorption and the generation of lipid signals in the gut, the utilization of absorbed choline and ethanolamine for mucosal and hepatic phospholipid synthesis and the acylation of absorbed lyso-PC with polyunsaturated fatty acids to chylomicron and mucosal phospholipids are important.

## Introduction

Polar lipids account for a small proportion of milk lipids. They are located mainly in the milk fat globule membrane (MFGM). With two of its three membrane layers originating from the apical membrane of the mammary gland epithelial cells, the MFGM has features of the plasma membrane lipid composition (1). Phosphatidylcholine (PC), sphingomyelin (SM), diacyl- and plasmalogenic phosphatidylethanolamine (PE) are major polar lipids. In addition, MFGMs contain phosphatidylinositol (PI), phosphatidyl serine (PS), and glycosphingolipids. A major difference in phospholipid (PL) composition compared to commonly used milk replacement formulas is the high content of SM.

Milk fat globule membrane–enriched formulations may now be used in milk-replacement formulas and food additives. Potential health benefits may be related to choline, ethanolamine, and polyunsaturated fatty acid (PUFA) content effects in the gut mucosa (2), an inhibitory effect on cholesterol absorption, and an influence on the gut microbiome and immune function (3). Recent reviews have summarized the potential health effects of milk PLs (4), its effects in the gut of the neonate (5), and the enzymes involved in the digestion of choline PLs (6).

Here, we summarized the pathways for the digestion and absorption of milk polar lipids. We linked these pathways to the potential biological effects in the suckling neonate and the adult who ingests larger doses of MFGM lipids in dairy products. Our starting point was a long-term interest in the digestion and absorption of sphingolipids and other polar lipids and in chylomicron metabolism.

## Composition of Polar Lipids in Milk

### Polar Lipids in Milk

Lipids in milk are first aggregated in droplets in the endoplasmic reticulum of lactating cells in a bleb or bud and then released in the alveolus lumen. During formation and secretion, the droplets fuse with each other and grow in size. At this stage, the droplets are enclosed in a single layer of membrane, and the main composition inside is triacylglycerols (TAG). At the surface of the lactating cells, these droplets are enveloped by two layers of plasma membrane and form the so-called milk fat globule membrane (MFGM). They are then secreted. The last two layers are enriched with PLs and sphingolipids. The polar lipids in the milk are, therefore, integral constituents of the globule membrane (1, 7).

Polar lipids account for about 1% of milk fat. The milk fat globules have a diameter ranging from 3 to 5 μm. In the MFGM, a variety of PLs, glycosphingolipids, and cholesterols associate with membrane proteins. The design and function of the MFGM, which is similar in humans and other mammals, is to stabilize the emulsion in the serum phase of milk and maintain permeability that facilitates digestion in the newborn. The composition of the PLs differs slightly among species. The total amount of PLs in the raw milk of a cow is 29.4–40 mg/100 g and in human milk, approximately 10–40 mg/100 g (4, 8).

The predominant PLs in both human and bovine milk are SM, PC, and PE. PS and PI account for smaller fractions (9) (Table 1). PE, PI, and PS are enriched in the inner surface layer of the MFGM, whereas most of the PC, SM, and glycosphingolipids are located in the outer MFGM bilayer. The fatty acid composition of SM is dominated by long and very long-chain saturated fatty acids as 16:0, 22:0, the uncommon 23:0, and 24:0. Phosphatidylcholine contains a high concentration of saturated fatty acids and of oleic and linoleic acid. In human but not in bovine milk, PE is a source of arachidonic acid (ARA) and docosahexaenoic acid (DHA). α-linolenic acid C18:3 can be found in small amounts in both human and bovine milk, with the content being influenced by diet (18). The acyl chains in human PLs are, thus, more unsaturated than in PLs of bovine milk. The PL content of bovine and human milk is compared in Table 1.

**Table 1:** Amounts of phospholipids (PLs) in human and bovine milk (mg/100 ml).

**Phospholipids**	**Human (mg/100 mL)**	**% of total PL**	**Bovine (mg/100 mL)**	**% of total PL**
SM	6.8–10.3	28.5–43.3	5.2–11.7	4.0–29.5
PE	5.2–9.9	21.8–41.6	6.9–18.7	23.2–72.2
PC	2.6–6.0	10.9–25.2	5.6–13.3	8.0–46.4
PS	0.8–4.3	3.4–18.0	0.6–3.6	3.4–24.5
PI	0.7–4.0	2.9–16.8	1.2–3.6	1.4–7.5

*Data on mg/100 ml in human milk are from Duan et al. (10), Giuffrida et al. (11), Thakkar et al. (12), Wei et al. (13), Ma et al. (14), Claumarchirant et al. (15), and on bovine milk from Pimentel et al. (16) and Graves et al. (17). Data on % distribution in human milk are from Giuffrida et al. (11) and, in bovine milk, are from Anto and Warykas (4)*.

The phospholipid content in human milk corresponds to a daily intake of 140 mg in a 4-week-old breastfed infant. The total choline intake is about 125 mg/day. Water-soluble choline compounds such as glycerophosphocholine, choline phosphate, and free choline account for about 80% of the choline content in milk and SM and PC for about 20% (19). Milk also contains water-soluble forms of ethanolamine. A recent review states that the concentration of ethanolamine in the blood and breast milk is 2 μM (range of 0–12 μM) and approximately 46 μM, respectively (20). Some of the milk PE is plasmalogenic PE, and human milk is rich in ARA and DHA (21). The PI is rich in PUFAs but accounts for only a small fraction of the total inositol in milk.

### Polar Lipids in Dairy Products

Dairy products vary in proportion between TAG and polar lipids. Butter, some cheeses, and cream are the most TAG rich, and their coproducts buttermilk and whey are relatively enriched in PL. The content of polar lipids in whole milk is about double the amount in skim milk. Lipids in buttermilk contain about 12% polar lipids, which can be more concentrated using ultrafiltration (22, 23). Butter serum is obtained after the melting and centrifugation of butter, and thus contains a slightly higher proportion of polar lipids than butter milk. Studies have shown the health effects of the intake of polar lipid-containing products; thus, the dairy industry has developed formulas even more enriched in such lipids (4).

## The Digestion of MFGM

### Triacylglycerol Digestion

The hydrolysis of milk TAGs in the suckling newborn depends mainly on the combined action of gastric lipase, the milk and pancreatic bile salt stimulated lipase (BSSL), and the pancreatic lipase-related protein 2 (PLRP 2), which are all expressed at birth (24). The digestion of TAGs is initiated in the stomach, where the MFGM becomes less stable at low pH, pepsin digests protein in the membrane, and gastric lipase gets access to TAGs (24). This enzyme is specific for the Sn 3 position and hydrolyzes medium chain fatty acid esters faster than long-chain fatty acid esters (25). Gastric lipase was found to hydrolyze bovine milk PL-emulsified lipid droplets more effectively than soy lecithin-emulsified lipid droplets with a similar size (26). In newborn pigs, lipid digestion and absorption were most effective with a formula using an MFGM-derived emulgator (27). Although the intestinal epithelium is differentiated, the digestive and absorptive capacity of the pancreas and small intestine is rapidly developing after birth, and the bile acid levels in the gut are lower than in the adult (28). The fatty acid absorption is incomplete (29). Although the average length of the small intestine is 275 cm at birth, i.e., half the average adult length, the tissue mass of the small intestine, the secretion of bile salts, and bile PC and the pancreatic secretory capacity expand rapidly after birth (28, 30). Although the development of the brain is the interest in focus for the in neonatal PL metabolism, the utilization of PUFAs and nitrogen bases in milk for expansion of the intestinal PL pools and hepatic PL traffic during maturation of the absorptive capacity must be large (28, 31–33).

**Figure 1:**
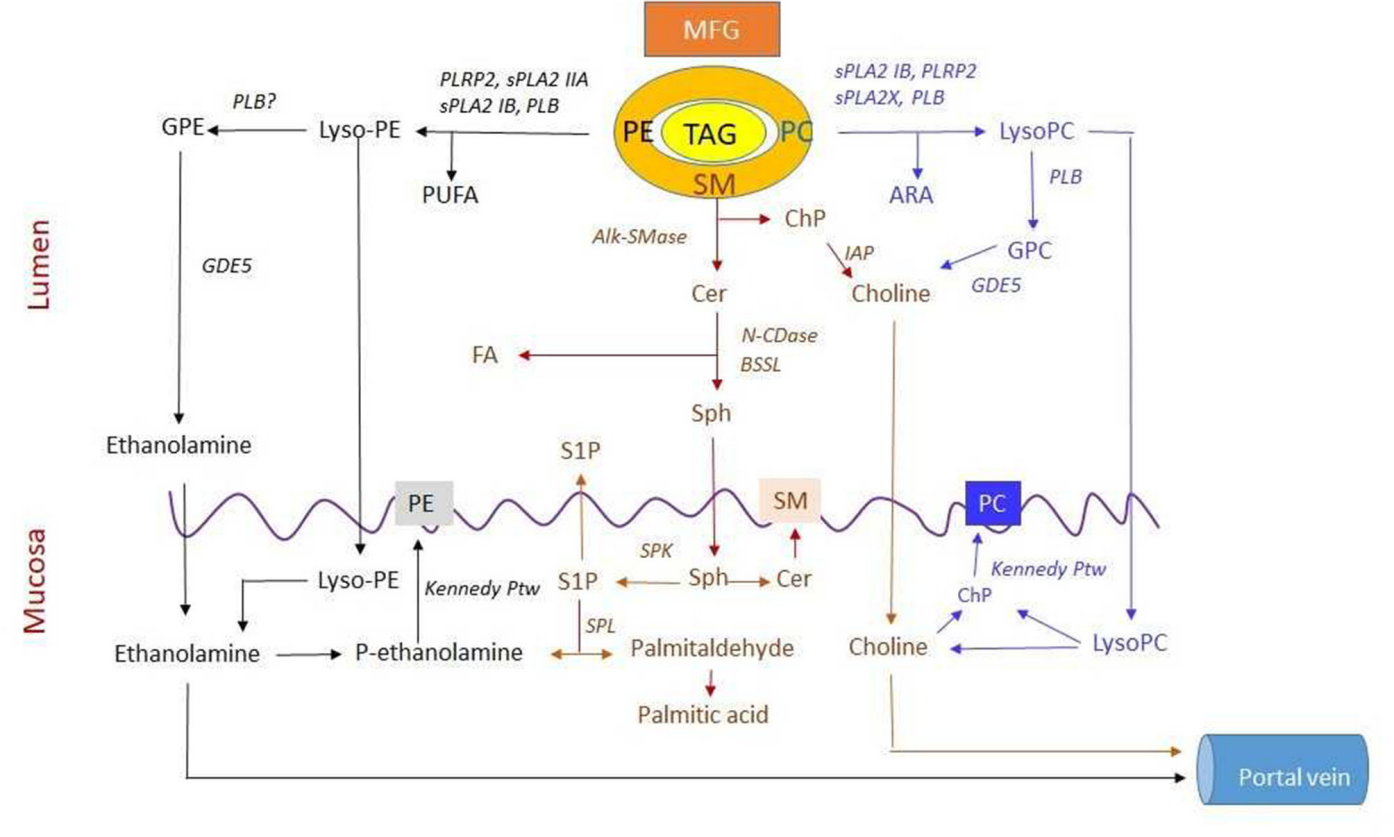
Digestion and absorption of three major polar lipids in milk. Lipids in the milk are organized in the milk fat globule (MFG) with polar lipids in the membrane and triacylglycerol (TAG) in the core. The secretory pancreatic phospholipase A2 IB (sPLA2 IB), sPLA2, and sPLA2X hydrolyze phosphatidylcholine (PC) to lysoPC and may release arachidonic acid (ARA) for both phospholipid (PL) and prostaglandin (PGD) synthesis. Phospholipase B (PLB) can degrade both PC and lysoPC to glycerophosphocholine (GPC) and then by GDE5 to choline. Both lysoPC and choline are absorbed. Absorbed lysoPC may release choline that can, in turn, be phosphorylated to choline phosphate (ChP) in mucosal cells. Sphingomyelin (SM) is sequentially hydrolyzed by alkaline SMase (alk-SMase), neutral ceramidase (N-CDase), and bile salt-stimulated lipase (BSSL) to ceramide (Cer), sphingosine (Sph), and fatty acid (FA). The cleaved ChP is degraded to choline by intestinal alkaline phosphatase (IAP). Then, Sph, choline, and FA are absorbed. The Sph in mucosa may be phosphorylated by sphingosine kinase (SPK) to S1P, most of which is degraded by sphingosine lyase (SPL) to palmitaldehyde and further converted into palmitic acid. This reaction generates ethanolamine phosphate (ethanolamine-P). Both ethanolamine and choline are substrate for synthesis of phosphatidylethanolamine (PE) and PC, respectively, via the Kennedy pathway or transported via portal vein. PE is degraded by sPLA2 IB, sPLA2 IIA, PLRP2, and PLB to lysoPE for absorption. LysoPE can be degraded by PLB to glycerophosphoethanolamine (GPE) and then to ethanolamine likely by GDE5 for absorption and resynthesis of PE. Meanwhile, polyunsaturated fatty acid (PUFA) can be formed during PE hydrolysis. Furthermore, transport and metabolism of PUFA from PE, FA from SM, and ARA from PC hydrolysis are not shown in the figure (For other abbreviations, see the text).

Even in adults, the gastric lipase contributes to fat digestion. Emulsification of milk fat may enhance lipolysis and thereby cause an earlier peak in the post-prandial TAG level (34). In adults, duodenum and jejunum TAGs are effectively hydrolyzed through the concerted action of pancreatic colipase-dependent lipase (Lip-Col) and pancreatic BSSL. Lip-col acts effectively at an oil/water interphase where bile salts stabilize the colipase/lipase binding (35). It is a high-capacity enzyme that confers the ability to digest large fat-rich meals. The action of the enzyme on PC-stabilized TAG Emulsions in the presence of physiological bile salt concentration exhibits a delay but is enhanced through the initiation of PC hydrolysis by the secretory pancreatic phospholipase A2 IB (sPLA2 IB) (36). Lip-Col and sPLA2 IB interact effectively in the hydrolysis of milk fat globule TAGs (37). The enzymes that participate in TAG, glycero-PL, and SM-digestion are summarized in Figure 1.

### Glycerophospholipid Digestion

As recently reviewed (6), the effective hydrolysis of the 2-ester bond of PC by sPLA2 IB is highly influenced by the bile salt/PL ratio. Although much of the PC hydrolysis by this enzyme coincides with TAG hydrolysis, part of the PC digestion is more extended. PL hydrolysis in a bile salt/PL/TAG emulsion occurs mainly in PL-rich bile salt/PL micelles that are formed as the TAG and PL hydrolyses proceed. As these micelles shrink in size due to the absorption of fatty acids and monoacylglycerols and the hydrolysis of some PLs, the sPLA2 activity against remaining PC declines with an increasing bile salt/PC ratio. We, therefore, believe that there are both pancreatic and mucosal phases of PC digestion that are mediated by the combined action of sPLA2 IB and PLB (6). The mucosal phospholipase B (PLB) (38), which is expressed in the mucosal brush border, particularly in the lower jejunum and ileum, hydrolyzes both ester bonds of PC even at high bile salt/PC ratios. This PLB is a protease-resistant and bile salt-dependent enzyme that may act throughout the whole lower part of the small intestine and must have an important role in the completion of the PC digestion [for ref see Nilsson and Duan (6)]. It acts both at the brush border and in the lumen. Studies on PLB knockout mice are, however, so far lacking. Even sPLA2 X is expressed in the mucosa and may have a role in the mucosal phase of PC digestion (39).

There is a lack of studies regarding the dietary effects of PC digestion. Pancreatic sPLA2 IB is a high-capacity enzyme; the activity of which depends on the interfacial properties of substrate and bile salt concentration. Furthermore, PLB has broad substrate specificity and hydrolyzes even glycerolipids and retinol esters. Dietary factors and the course of lipid digestion are, therefore, expected to influence the two enzymes differently. Interestingly, in a mouse, a fat-free vs. a fat-rich diet did not influence levels of pancreatic sPLA2 IB or mucosal PLB, but, when experimental chronic pancreatitis was developed, the decrease in sPLA2 IB was compensated for by an increase in PLB (40).

*In vitro*, phosphatidylethanolamine is hydrolyzed by PLA2 over a wide range of bile salt/PL ratios and a mix of PE and PC is more effectively hydrolyzed than is pure PC (41). Furthermore, PLRP2 has a higher activity against PE than against PC (42). *In vivo*, glycerophophorylethanolamine and ethanolamine are also formed in the lumen (43), and PLB is also active against PE (38). *In vitro*, human pancreatic PLA2 IB also hydrolyzes PI as effectively as PC and with the same biphasic influence of bile salts (44) (Figure 1).

The intestine also produces a sPLA2 IIA, which originates from the Paneth cells and does not attack PC but is active against PE, a dominant PL in gut bacteria (45). Since this enzyme is bactericidal, (46) it may have a dual function of preventing the utilization of ethanolamine by bacteria and recovering ethanolamine from dead bacteria and sloughed mucosal cells in the lower small intestine.

Of the milk phosphatidylethanolamine, some is plasmalogenic. Although PLA2 IB hydrolyzes the 2-ester bond, there is no hydrolysis of vinyl ethers in the gut lumen. The reacylation of acyl- and plasmalogenic lyso-PE into chylomicron PE is rather small. Plasmalogenic lyso-PE is degraded in the mucosa [for ref see Nilsson and Duan (6)] by the intracellular intestinal lysoplasmalogenase, which has been cloned and characterized, and which is found at its highest levels in the jejunum and upper ileum (47).

In humans, sPLA2 1B is not expressed at birth (48). In human neonates, PLB is poorly studied; but, in rabbits, it is expressed post-weaning (49, 50). Additionally, in mice, PLB and sucrase-isomaltase, which is also expressed late, share similar enterocyte specific promoter structures (51). This speaks against a role in neonatal PC digestion in these species; yet meconium from humans contains both PLA2 activity and phospholipase activities, which hydrolyze PC to fatty acids and glycerophosphocholine (52, 53).

Thus, PLRP2, which is active against both PC and PE, may be essential for the milk glycero-PL hydrolysis in newborns (42). Furthermore, BSSL has broad substrate specificity. It has some activity against short-chain PC and lyso-PC under some *in vitro* conditions but is not active against long-chain PC and lyso-PC in the presence of physiological concentrations of bile salts (54) [for other ref see Nilsson and Duan (6)]. Since BSSL hydrolyzes lyso-PC in the absence of bile salts, the question on whether it may hydrolyze lyso-PC in neonates where bile salt levels are low is still relevant.

It is unknown when the expression of sPLA2 IB increases after birth. One might expect that it occurs when the bile acid pool expands and the bile PC secretion increases soon after birth, but data are lacking. Data on the course of digestion of glycero-PLs in newborns are scarce. When 11 duodenal contents from suckling infants, <1 month old, were analyzed for polar lipids, PE concentration was found to be very low in all samples. Lyso-PE dominated. The lyso-PC concentration exceeded the PC concentration, but a considerable amount of unhydrolyzed PC remained (55). These few data indicate that, even in the neonate, PE is rapidly digested in the proximal gut and PC digestion is more extended.

### Sphingolipid Digestion—Enzymes Involved

#### SMase

SM is not hydrolyzed by any pancreatic enzymes but by sphingomyelinase (SMase), which cleaves the phosphocholine head group from SM and turns it into ceramide. Ceramide, in turn, is hydrolyzed by a ceramidase that cuts the acyl chain and turns ceramide into sphingosine for absorption. Based on the optimal pH, three types of SMases called acid, “neutral,” and “alkaline SMases” have been identified. The key SMase responsible for digestion of SM in the gut is alkaline SMase (alk-SMase), which was discovered in the intestinal mucosa by Nilsson (56). Further studies show that it is attached to the surface of the intestinal brush border by a short intracellular domain with its active catalytic site exposed in the intestinal lumen (57). Alk-SMase shares no sequence similarities with either acid or neutral SMase but belongs to the ecto-nucleotide pyrophosphatase phosphodiesterase (eNPP) family (58). As a new member in the NPP family, alk-SMase is also called NPP7. Other enzymes in the NPP family hydrolyze nucleotides and nucleosides and are involved, e.g., in purinergic signaling, and autotaxin (NPP2) generates lysophosphatidic acid from lyso-PC and has important signaling functions related to inflammation, cell migration, and tumorigenesis (59, 60).

Although located on the surface of the brush border, alk-SMase can be released into the intestinal lumen by bile salts and by trypsin, which cleaves the short intracellular domain of alk-SMase (61). Both the brush border-bound form and the released form are active, with the activity of the free form being higher than the mucosal form. The optimal pH of alk-SMase is approximately 9, but it becomes active at pH 6 and above (62). A striking property of the enzyme is its resistance to pancreatic proteases. Its activity can be identified along the whole intestinal tract and in the stool (62). The activity in the fecal samples reflects the capacity of SM digestion of an individual. In alk-SMase KO mice, SM in the intestinal content was increased 6-fold and, in the feces, this increase was by 243%; whereas ceramide in feces decreased by 74% (63). Alk-SMase is bile salt dependent. Taurocholate (TC) and taurochenodeoxycholate (TCDC) strongly enhance their activity at their critical micelle concentration (CMC) (57). In animals, alk-SMase activity is not found in the stomach, is very low in the duodenum, increasing in jejunum and ileum, and declining in the colon. The considerable alk-SMase activity was unexpectedly found in the hepatic bile of human beings, but not in the bile of any other mammalian species (64, 65). The bile alk-SMase is most likely localized on the surface of the canalicular membrane and released by bile salts into the bile. The secretion of alk-SMase in the human bile may endue humans with a higher capacity to digest dietary SM, and the digestion can start earlier in the duodenum.

Studies on fetal rats showed that the expression of alk-SMase sharply increased during day 20–23 of gestation (66). At this stage, the gut epithelium undergoes rapid differentiation with the formation of mature villus cells, distinct villus, and crypt structures soon before birth at day 23 of gestation. Studies on 3-week-old suckling pigs also showed high levels of alk-SMase in the jejunum and ileum (55). Furthermore, significant levels of alk-SMase were found in the meconium from both preterm and term human infants (67). Put together, the data indicate that nature endues the newborn mammals with the capacity to digest the SM in the breast milk after birth.

Alk-SMase can also cleave cholinephosphate from PC and lysoPC. The activity against PC is weak, about 2–8% of that against SM (57, 65). The activity against lysoPC is greater than against PC, but is still much lower than against SM. Differing from the activity against SM, the optimal pH for alk-SMase to hydrolyze PC and lyso PC is 7.5. The formed cholinephosphate is a good substrate for intestinal alkaline phosphatase (68).

Acid and neutral SMase are two other SMases that hydrolyze SM to ceramide at different optimal pHs. There are two forms of acid SMase translated from the same gene SMPD1 but vary in glycosylation: One form is localized in the lysosomes intracellularly. The other form is secretable, and its activity can be identified in several body fluids, including the salivary and intestinal contents (69, 70). Acid SMase activity was found in milk, which hydrolyzes milk SM *in vitro* in the presence of bile salt (71), but no ceramide formation was seen in gastric contents from newborns (55). In addition, a relatively low activity of neutral SMase has been identified in the intestinal content, which is probably derived from the shedding of mucosal cells. The contribution of neutral SMases to milk SM hydrolysis is probably not significant because, differing from alk-SMase, they are rapidly inactivated by pancreatic enzymes in the intestinal tract (69). That is why, in alk-SMase KO mice, more than 90% of given SM accumulated in the gut (63).

#### Neutral Ceramidase and Bile Salt-Stimulated Lipase

In general, long-chain ceramides formed by SMase are poorly absorbed and are further hydrolyzed by neutral ceramidase (N-CDase) to sphingosine and fatty acids, which are readily absorbed (72). The optimal pH of N-CDase is 7.6. This enzyme was purified from rat (73) and human (74) intestinal tracts. The amino acid sequence shows that the intestinal N-CDase is the product of the *Asah2* gene and has been previously cloned in the liver, kidney, and other tissues (75–77).

Similar to intestinal alk-SMase, the intestinal N-CDase is an ectoenzyme that is attached to the surface of the intestinal mucosa with a hydrophobic domain at its N terminal. The active site of the enzyme is exposed in the intestinal lumen, which facilitates its function to hydrolyze ceramide formed in the lumen by alk-SMase. N-CDase is also easily released from intestinal mucosa by bile salt. The activity of N-CDase also requires the presence of bile salt. The levels of both enzymes are significantly decreased in bile-diverted rats (78). Similar to alk-SMase, N-CDase is resistant to pancreatic proteases, such as trypsin and chymotrypsin. The enzyme can be transported along the whole gastrointestinal tract to the colon in active forms and can also be identified in the feces.

As mentioned, BSSL in milk and pancreatic juice have a key role in TAG digestion in both neonates and adults. In particular, BSSL was also found to hydrolyze the amide bond between sphingosine and fatty acids in ceramide (71). The activity is bile salt dependent, and the optimal pH is approximately 8.5. There is, however, little ceramide formation in the proximal gut where BSSL is most active. In addition, ceramide digestion was normal in BSSL KO mice (55) but was inhibited in N-CDase KO mice (79). Based on these findings, it can be concluded that N-CDase is the most important enzyme in ceramide digestion in the intestinal tract.

### The Capacity of SM Digestion

As summarized, (80) alk-SMase and N-CDase act mainly in the middle of the small intestine. The digestion of SM is more extended than that of TAG and is dose dependent. When up to 25-mg SM was given orally to rats, the course of digestion became more extended with an increasing dose (81). In ileostomy patients feeding up to 200-mg milk SM in a test meal generated only modest increases of some ceramide and SM species in the content (82). A recent study on post-menopausal women who ingested cream cheese with 3- or 5-g milk polar lipids for 4 weeks found that the ceramide content in feces and ileostomy increased more than the content of milk SM species (83). The conclusion from these studies is that SM is extensively digested even with an MFGM lipid load, whereas ceramide digestion is more incomplete.

The course of digestion of SM in the neonate is unknown, although alk-SMase and N-CDase were found in the meconium of both preterm and term human infants (67). In duodenal content from babies <1 month old (55), no significant hydrolysis of SM to ceramide was observed. Thus, SM digestion is likely to be more extended than the PC and the PE digestion in the neonate. The digestion of SM has not been studied in newborn NPP7 KO mice, although these mice grow normally during suckling and exhibit a normal gross phenotype (63).

### Factors Affecting SM Digestion

#### Enzyme Levels in the Gut

Many factors may affect the efficiency of milk SM digestion. Obviously, the level of alk-SMase is important since, in alk-SMase KO mice, the major part of orally given radiolabeled SM was recovered in feces, and the appearance of radioactive SM fatty acid was reduced by 95 % (63). A human study indicates that the level of alk-SMase is generally stable but may decrease somewhat above 45 years of age, indicating that the efficiency of SM digestion may decline in aged persons (84).

Dietary factors affect alk-SMase expression. The continuing increase of alk-SMase activity after birth in newborn rats indicates that SM in the milk may upregulate alk-SMase expression. Feeding adult ICR mice with SM for 22 weeks increased alk-SMase by 65% in the colon with increased enzyme protein and mRNA (85). Feeding rats with a water-soluble psyllium fiber for 4 weeks increased alk-SMase activity in the colon about 2-fold, whereas feeding high-fat diet decreased alk-SMase activity by about 75% (86). Insoluble fiber had no effect.

#### Bile Salts in the Gut

The activity of alk-SMase is bile salt dependent. All bile acids in the gut had some stimulatory effect on alk-SMase activity. The maximal effect occurs at their CMC. However, the stimulation by TC and TCDC, i.e., the taurine conjugates of the two primary bile acids, was 6- to 30-fold higher than the effect of glycine conjugates and of the secondary bile acid taurodeoxycholate (57). In human newborns, TC and TCDC are predominant bile acids. The mechanism for this type-specific activation of alk-SMase is not clear, but it is not due to a non-specific detergent action as non-physiological detergents, such as Triton X100 and CHAPS, did not stimulate but inhibit the TC and TCDC-induced activation of alk-SMase (57, 58). A specific interaction between the enzyme protein, the lipid substrate, and bile salts must be involved. In the case of N-CDase, TC and glycocholate stimulated the activity similarly. N-CDase is also activated by non-physiological detergents, such as Triton X100, which abolishes alk-SMase activity, indicating that different mechanisms are involved.

In line with the finding that bile salts release and activate alk-SMase and N-CDase activities, the alk-SMase activity was decreased by 85% in the intestinal lumen and by 68% in the feces of bile-diverted rats (78). Bile salt deficiency may thus reduce the efficiency of SM digestion, e.g., in cholestatic disorders and short bowel syndrome.

#### Lipids in the Gut

Digestion of SM is influenced by the presence of other lipids in the gut. *In vitro* studies with purified alk-SMase in the presence of bile salts showed that hydrolysis of SM is inhibited by other polar lipids, including PC, PE, and PI and lyso-PC (87). Moderate concentrations of long- and medium-chain free fatty acids stimulated the alk-SMase with lauric acid being most effective, whereas the inclusion of non-polar lipids as di- and triacylglycerols and cholesterol inhibited to a varying degree (88). Cholinephosphate was stimulatory (65). Even if alk-SMase is secreted in the bile in humans, one would thus expect that other lipids in the upper small intestine would delay the SM hydrolysis until after the peak of the TAG hydrolysis in the upper jejunum. Furthermore, the extended course of part of the PC hydrolysis and of the cholesterol absorption may influence the SM hydrolysis. The finalization of the PC hydrolysis by mucosal PLB and the hydrolysis of most of the SM by Alk-SMase may coincide in the jejunum and upper ileum (6). The SM digestion must be influenced by the course of digestion and absorption of other lipids, including other PLs and cholesterol.

## Effects of Dairy Polar Lipids on Cholesterol Absorption

### Mechanisms and Animal Studies

Cholesterol is highly hydrophobic and dependent on micellar solubilization for absorption. Dietary and bile cholesterol is embedded in the mixed micelles formed by dietary lipids and the PC-rich bile. As lipolysis proceeds, the mixed micelles will contain free fatty acids, monoacyglycerols, and lyso-PC, as well. The course of both TAG and PC digestion will influence the absorption of cholesterol from such continuously changing mixed micelles (89, 90). The backbone sphingoid base of SM increases the polarity of SM and allows for stronger intermolecular hydrogen bonding between cholesterol and SM compared with other PLs (91, 92). The effect is mutual. Cholesterol slows down the rate of SM micellization with bile salts (93) and inhibits SM digestion (94). Cholesterol is transported across the enterocyte cell membrane by transmembrane-carrier proteins Niemann-Pick C1-like 1 (NPC1L1), CD36, and SR-BI, which are pivotal transporters that are located at the apical membrane of the small intestine (95). The cholesterol absorption is incomplete because cholesterol is also expelled from the mucosal cells by the ABCG5/G8 transporter (95).

After the initial observations that SM inhibits cholesterol absorption *in vivo* in rats (94), in lymphatic duct cannulated rats (96), and in Caco 2 cells (97), several animal studies have shown decreased cholesterol absorption after intake of milk SM (98, 99). In mice, milk SM and the products ceramide and sphingosine reduced plasma and liver lipids, including cholesterol (100).

Digestion of SM enhances cholesterol absorption since this was decreased in alk-SMase KO mice (101), indicating that the physical interaction between cholesterol and SM is essential for the inhibition of cholesterol absorption by SM. On the other hand, the generation of ceramide by alk-SMase increased the SM-induced inhibition of cholesterol absorption in the Caco 2 cell model. In this model system, the ceramide is not further digested (102). Long-chain ceramides and cholesterol have similar hydrophobicity. Both have low solubility in bile salt micelles without any other amphiphilic lipids. As fatty acids, monoacylglycerols, and polar lipids are absorbed, remaining ceramide and cholesterol may be poorly solubilized. This might explain why the absorption of the ceramide part of SM is more incomplete than the digestion of SM. Yet much of the ceramide is hydrolyzed *in vivo*, and the net effect of SM hydrolysis is expected to favor cholesterol absorption under most conditions.

A recent study finds that the NPC1L1 knockout and ezetimibe inhibit not only the absorption of cholesterol but also of the choline part of SM (103). It was assumed that the NPC1L1 transporter is an alternative pathway for SM absorption. Another possible explanation is that the inhibition of cholesterol absorption may increase luminal interactions between cholesterol and SM. Nevertheless, absorption of some intact SM and ceramides into the epithelial cell is possible since the absorption of SM in the alk-SMase KO mice is, although significantly decreased, not zero. After feeding radiolabeled ceramide or SM, a small proportion was found as ceramide in chyle (72) and specific milk SM ceramide species were found in chyle after feeding milk SM. Interestingly, this amount was increased when other polar milk lipids in addition to SM were included (104). The hydrolysis of glycero-PLs and other glycerolipids is necessary for the effective absorption of cholesterol and fat soluble vitamins via NPC1L1, CD36, and SR-B1 (105). Although glycero-PLs may inhibit SM and ceramide digestion as described above, the digestion of these lipids might also enhance the uptake of intact SM and/or ceramide. The effect of different lipid vehicles on sphingolipid digestion and absorption needs to be further studied *in vivo*.

### Human Studies

Human studies indicate that MFGM-rich products reduce cholesterol absorption, although results vary. Conway et al. (106) found that buttermilk consumption decreased cholesterol absorption and lowered plasma cholesterol. On the other hand, Ramprasath et al. (107) found no effect on cholesterol absorption, metabolism, or plasma lipoproteins with the ingestion of 1-g milk SM per day for 2 weeks, and Keller et al. found some cholesterol-lowering effect but no beneficial change in the HDL/LDL cholesterol ratio when effects of 3- and 6-g milk polar lipids per day were studied over a 10-day period (108).

On one hand, a single dose of 1 g of milk polar lipids given to healthy individuals had little to no effect on post-prandial plasma lipids (109). On the other, a comprehensive study on post-menopausal women and subjects with ileostomy showed a reduced level of post-prandial TG-rich lipoproteins after a 4-week daily intake of 5-g dairy-PL in the form of cream cheese (98). The same study also demonstrated that milk PL decreased the incorporation of dietary cholesterol into chylomicrons. In ileostomy subjects, both cholesterol and SM were increased in the ileal fraction after intake of milk PL. In a second study, it was concluded that the 4-week milk PL supplementation (both 3 and 5 g) decreased post-prandial total cholesterol, apoB/apoA1 ratio, total chylomicron SM and ceramide, and also the levels of potentially atherogenic SM and ceramide species (83).

## Postabsorptive Metabolic Pathways

### The Fate of Lyso-PC and Choline in the Mucosa

The gut epithelium contains high levels of sphingolipids, PC, PE, and plasmalogenic PE. In particular, PC is needed for an effective secretion of chylomicrons [for ref see (6, 110, 111)] and for the secretion of surfactant in the small intestine, which is rich in saturated PC species (112). Fat intake per body weight in newborns (2.5–3.5 g/kg/day) is three to five times higher than in adults. A considerable amount of PC is needed for the secretion of chyle lipoproteins. However, data on the PL content in chyle chylomicrons of the human neonate are lacking. In 2-week-old suckling rats, the PL/TAG ratio was about 6-fold higher in chylomicrons and manifold higher in total chyle lipoproteins than in rat milk (113).

Reacylation into chylomicron PC of absorbed lyso-PC formed by the action of PLA2 IB on dietary and bile PC increases with the dose of ingested fat (114). In adult humans, the amount of PC secreted in the bile significantly exceeds the amount of ingested PC [for ref see Nilsson and Duan (6)]. However, quantitative data on bile PC secretion in suckling human neonates are difficult to find. In mice, the hepatic ABCB4 transporter that mediates the transfer of PC into the bile is expressed just at the time of birth (115). The PL concentration in the bile in 2-week-old suckling rats was as high as 11.8 mM, and the ratio of PL/bile salts was similar to that in adult rats (113).

Different fatty acids are asymmetrically distributed between TAG and PLs in chyle, with the PUFAs and saturated fatty acids being overrepresented at the 2- and 1-position of the PLs. After feeding labeled ARA or linoleic acid in cream to lymphatic duct-cannulated rats, both the radiolabels and the masses of these two PUFAs were higher in PLs than in TAG (116). The retention of ^3^H-ARA in mucosal PLs was high (about 25% after 4 h). ^3^H-ARA retention in mucosal PE and PI was severalfold higher than that of ^14^C-LA (117). Selective use for PL formation in the absorptive mucosa is thus a major pathway for absorbed ARA, whether it comes from milk PE or TAG, other dietary sources, or bile PC (118).

Among the enzymes that mediate the selective reacylation of lyso-PLs (119), the gut lyso-PC acyltransferase 3 (LPCAT 3) is crucial to the acylation of ARA and other PUFAs into mucosal and chylomicron PC. In mice, the genetic deletion of this enzyme results in lipid accumulation in the mucosal cells and malnutrition (110, 111, 120). Oral administration of PC and olive oil allowed the LPCAT3 KO mice to survive, but the KO mice had shorter and wider small-intestinal villi and a larger small intestine. Plasma membranes of the enterocytes from LPCAT3-deficient mice also had reduced levels of NPC1L1, CD36, and FATP4, i.e., proteins that regulate lipid acid uptake, and a reduced ARA level in brush border PLs. The uptake of lipids by the small intestine was reduced.

During fat absorption, PC synthesis in the mucosa increases. Kennelly et al. (121) found that the selective intestinal deletion of CTP-phosphocholine cytidyltransferase α (CCT-α), the isoform of CCT that catalyzes the major regulatory step in the *de novo* synthesis of PC, decreased mucosal PC content but did not influence chylomicron formation. When a high-fat diet was used, however, a decreased uptake of fatty acids was observed. There was a compensatory increase of bile PC, related to a more proximal absorption of bile acids and increased enterohepatic bile acid secretion. Mucosal PC synthesis has such important metabolic implications.

The gut intestinal epithelium also secretes highly saturated molecular species of PC into the gut lumen (122), analogous to the secretion of lung surfactant. The quantity is difficult to estimate, and it is unknown whether the acylation of lyso-PC with a saturated fatty acid contributes to this secretion [for ref see Nilsson and Duan (6)].

The bacterial generation of trimethylamine and the subsequent generation of potentially harmful trimethylamineoxide from choline in the gut has received great attention (123). As recently demonstrated (124) and summarized (6), the rapid absorption of generated lyso-PC and the effective digestion by PLB may lead to less trimethylamine formation from choline in PC than from larger doses of orally given free choline.

In conclusion, mucosal reacylation reactions and *de novo* synthesis of PC are essential for normal lipid absorption and chylomicron formation. During suckling, the milk PC and SM contribute to these pathways, but other choline compounds in milk and bile PC are major contributors. Free choline transported by the portal vein is extensively used for hepatic synthesis of bile PC (81). Thus, there is a considerable indirect enterohepatic circulation of choline. Interference with this recirculation may be of crucial importance, e.g., in cystic fibrosis (125) and short bowel syndrome [for ref see Nilsson and Duan (6)]. Table 2 summarizes the pathways for the utilization of the choline part of milk-PC.

**Table 2:** Milk phosphatidylcholine (PC) and sphingomyelin (SM) are sources of choline that are used for mucosal PC synthesis or recycled via the portal vein and as chylomicron PC.

**Milk polar lipid and post-prandial choline and ethanolamine metabolism**	
**Event**	**Importance**
Milk SM and PC contribute but are not major sources of choline in the neonate	Milk PC and SM account for 20%, and cholinephosphate, glycerophophocholine and choline for 80% in human milk (19)
Reacylation of absorbed lyso-PC, mainly from bile PC, is a major source of chylomicron PC	Access of lyso-PC for reacylation determines maximal rate of production and PL/TAG ratio of chylomicrons (126, 127)
Use of absorbed choline for mucosal PC synthesis	Mucosal PC synthesis is necessary for normal mucosal function and lipid absorption (121)
A major part of the choline from PC and SM is transported as free choline by the portal blood	Effective uptake of choline by the liver and extensive use for PC synthesis. Newly synthesized PC is preferentially secreted as bile PC [for ref see Nilsson and Duan (6)]
Limited reacylation of absorbed lyso-PE and plasmalogenic lyso-PE into mucosal and chylomicron PE but active mucosal PE synthesis (43)	Reacylation not necessary for chylomicron production. Yet, nascent chylomicron PLs contain 8–12% PE (116)
Transport of ethanolamine by the portal blood (43)	Effective uptake by the liver and extensive use for hepatic PE synthesis (128)
Mucosal metabolism of absorbed sphingosine from milk SM generates ethanolamine	Supplies ethanolamine to the mucosa over a more extended part of the small intestine (81)
Course of PE and SM digestion minimize bacterial utilization of ethanolamine (129)	PE digestion and absorption is rapid in upper small intestine (43) and sphingosine generatesethanolamineintracellularly

*Supply of ethanolamine via phosphatidylethanolamine (PE) and via metabolism of the sphingoid bases of SM contribute to mucosal PE formation and the secretion of chylomicron PE, and to hepatic PE synthesis*.

### The Utilization of Lysophosphatidylethanolamine and Ethanolamine

Table 2 also summarizes the utilization of the ethanolamine. After the extensive degradation of PE in the proximal jejunum (43), rather small proportions of lyso-PE and plasmalogenic lyso-PE are reacylated into chylomicron and mucosal PE. Some ethanolamine is used for PE synthesis in the mucosa. After milk fat intake, PE transports significant proportions of the ARA and DHA in chyle (116). Of the ethanolamine that is transported *via* the portal vein, much is incorporated into hepatic PE (43).

Absorbed sphingosine is converted to sphingosine-1-phosphate (S1P) by sphingosine kinase. Most of the S1P is converted to palmitaldehyde by S1P-lyase and ethanolamine-phosphate (80). Each SM molecule thus generates one choline and one ethanolamine molecule. Since the course of digestion of SM in the gut is more extended than the absorption of PE, milk SM and PE may together supply ethanolamine to the mucosa in the whole small intestine. Ethanolamine has trophic and antioxidative effects on the mucosa (130). S1P is also an important signal substance that may be fundamental to paracrine antiapoptotic signaling and immunoregulation in the mucosa. The partitioning of S1P generated from ingested SM between irreversible degradation by S1P-lyase and paracrine secretion and action on epithelial and immunocyte S1P receptors are of great interest [for ref see (5)]. S1P is found in chyle, but the concentration is lower than in blood. Information on whether concentration in chyle increases after SM feeding is lacking (131). The small intestine expresses the enzymes necessary for synthesis of sphingosine, ceramide, SM, glucosylceramide, and more complex glycosphingolipids [for see Duan and Nilsson (132)]. Although the sphingolipid synthesis is active during the formation of differentiated villi, the incorporation of absorbed sphingoid bases into complex sphingolipids is small. Furthermore, although sphingosine and sphinganine are metabolized similarly, dienoic and hydroxylated sphingoid bases in plant sphingolipids may be less effectively metabolized, some being expelled by the ABCB1 transporter and some transferred to blood as ceramide (133, 134).

Ethanolamine is used by many gut bacteria both for oxidation and for synthesis of PE, which is a major PL in gut bacteria (135). Certain intestinal bacteria, including some pathogenic species, use ethanolamine as a carbon and/or N source with the aid of ethanolamine utilization proteins (EutR). Changes in ethanolamine availability may alter both the colonization of ethanolamine-using bacteria and the virulence of pathogenic bacteria. The Paneth cell phospholipase A2 IIA has high activity against PE and is bactericidal [for ref see (6)]. The rapid digestion of PE in the proximal gut combined with the intracellular ethanolamine generation from sphingosine seems well-suited to optimize the ethanolamine assimilation by the host with minimal bacterial interference.

### The Targeting of PUFAs to Phospholipids

The milk fat globule is an energy dense product that which fuels fatty acid oxidation. Medium- and short-chain fatty acids are preferentially oxidized or converted into ketone bodies in the liver. The fact that suckling babies have a mild ketosis all the time reflects that milk fat metabolism is optimized for glucose sparing even when energy intake is sufficient. In this context, the fatty acids that are needed for the anabolic expansion of tissue PL pools should not be oxidized but selectively targeted to tissue PLs. Table 3 summarizes the pathways by which this occurs.

**Table 3:** Selective targeting of milk polyunsaturated fatty acids (PUFAs) to mucosal and chyle phospholipids (PLs), the metabolism of chyle PLs by LCAT, hepatic lipase and hepatic uptake, and the utilization of PUFAs for the dynamic hepatic PL metabolism are important features.

**The selective targeting of milk PUFAs to tissue PLs**
1. Milk LA, ARA and DHA supplied in TAG, PC, PE and plasmalogenic PE are selectively partitioned to mucosal (117)and chylomicron PLs (116) by selective reacylation of lyso-PC, by preferential use of unsaturated diacylglycerol species in the *de novo* synthesis of PE, and by deacylation reacylation cycles (136)
2. Selective acylation of lyso-PC with PUFAs via LPCAT3 (110)is essential for chylomicron secretion and normal fat absorption, and a major source of chyle PC. Mucosal *de novo* synthesis of PC is not selective for PUFAs but essential for normal lipid absorption (121)
3. Little absorbed lyso-PE is recylated into chylomicron PE. Yet chyle lipoprotein PLs contain a significant proportion of PE that is rich in ARA and DHA (116)
4. Chylomicron PC, PE, PI and SM are transferred to HDL by phospholipid transfer protein during the metabolism of the chylomicronTAG by lipoprotein lipase (137)
5. PC in HDL is metabolized by LCAT, hepatic lipase and hepatic uptake of intact PC (138). LCAT may generate both saturated and polyunsaturated lyso-PC species (138)
6. Secretion of polyunsaturated lyso-PC from liver (139) and lyso-PC generated by LCAT is an important transport vehicle for choline and fatty acid to tissues, including Msfa-2 mediated transport of choline and PUFAS into brain (140) and placenta (141)
7. PE in HDL is metabolized by hepatic lipase which generates 2-acyl-lyso-PE, and by hepatic uptake of intact PE (142)
8. ARA and DHA esters in chylomicron-TAG partially resistant to lipoprotein lipase and enriched in chylomicron remnants and partitioned to the liver. Some ARA is resecreted in bile PC and is used for mucosal PL formation (118)

*Arachidonic acid (ARA) exhibits the highest degree of selective partitioning to these pathways*.

The preferential partitioning of ARA and other PUFAs to mucosal PC *via* LPCAT 3 has already been mentioned. The CDP-ethanolamine pathway prefers molecular species containing DHA or AA in the case of PE formation (128). Selective fatty acid acylation of PUFAs into distinct PLs is a saturable process. With higher concentrations of PUFA-acyl-CoAs available, an increased proportion is incorporated into TAG, e.g., with high LA access, LA-CoA competes with the AA- and DHA-CoA for incorporation into chyle PC and PE. A larger proportion of all three fatty acids is then partitioned to TAGs, although the preferential incorporation of ARA into PE and PI was rather unaffected (116). Thus, feeding LA- or DHA-rich formulations to neonates may decrease the partitioning of ARA to PLs.

### Metabolism of Chylomicron Phospholipids

The partitioning of PUFAs to chylomicron PLs influences their further metabolism. During the metabolism of chylomicrons, most of the PC, PE, PI, and SM are transferred to HDL *via* phospholipid transfer protein (137, 143). HDL-PC is metabolized mainly by LCAT, hepatic lipase, and uptake to the liver as intact PC *via* the SRB1 receptor and unknown mechanisms (138). The SR-B1 receptor in the liver also mediates uptake of SM into the hepatocytes. In humans, chylomicron PC, after its transfer to HDL, is metabolized without rapid equilibration with LDL,VLDL, and erythrocyte membrane PC (144). Any PC-PUFA that is used by LCAT to produce cholesteryl ester is also transported to the liver *via* lipoprotein receptors and SRB1 receptor-mediated cholesteryl ester uptake. The fatty acid composition of plasma cholesteryl esters both in humans and rats reflects mainly the action of LCAT.

Interestingly, human LCAT, which normally transfers the 2-fatty acid in PC, may use fatty acids derived from the sn-1-position of 2-ARA-PC or 2-DHA-PC (145). If so, the action of LCAT on HDL-PC, originating from chylomicrons, may generate both saturated 1-lyso-PC and 2-AA and 2-DHA-lyso-PC. However, Lyso-PC is also secreted from the liver by the hydrolysis of hepatic PC (139). The major portion of fatty acids in this lyso-PC is also polyunsaturated. The most prevalent PUFA is ARA, followed by LA (139). How this release of lyso-PC from the liver is regulated is poorly known.

The conclusion is that both LCAT action on chylomicron-derived PC and hepatic secretion must be quantitatively important sources of lyso-PC. They are also determinants of the fatty acid composition of lyso-PC.

### The Quantitative Role of lyso-PC in Choline and Fatty Acid Transport

Few studies have considered the quantitative aspect of the role of lyso-PC in choline and fatty acid and phospholipid transport. In rats, 1-palmitoyl-lyso-PC (146) and 2-ARA-lyso-PCA (147) are eliminated much faster than lipoprotein-PC after iv injection. Combining data from different studies, one finds that lyso-PC concentration in blood is 13–32-fold higher than the concentration of free choline in humans and approximately 50-fold higher in rats. In rats, the half-life for the initial slope of lyso-PC elimination was 6–11 min (146), which corresponds to a clearance of the order 3–5.6 μmol/kg/min. From data on the elimination of IV-injected 3H-choline (148) and on the plasma concentration of free choline obtained by others, one may calculate that the turnover of plasma-free choline in rats is of the order 0.7 μmol/kg/min. After injection of 2-ARA-lyso-PC, the liver also exhibited the highest uptake per organ weight. The uptake by the small intestine per g was about half and the uptake per g by the brain was about one-tenth of that in the liver (147). Thus, lyso-PC is an important carrier of both saturated fatty acids, PUFAs including ARA and DHA, and choline to tissues. In parallel, there is a transporter-mediated uptake of free choline, including high-affinity transporter, for acetylcholine formation in neurons (149–151).

A role of lyso-PC in the transfer of PUFAs as AA, DHA, and choline over the blood brain barrier has long been advocated (139, 147, 152–154). Recently, a role of the transporter mfsd 2a (from the major facilitator superfamily 2a, a previously orphan transporter) in the uptake of DHA-lyso-PC (140) has been established. This transporter is expressed in the endothelium of the blood–brain barrier of microvessels (140). Any dietary influence on the formation of different molecular lyso-PC species may hereby be linked to an influence on the brain supply of choline and PUFAs. Defect mfsd 2a has been linked to developmental defects of the brain (155).

### Lyso-PC Traffic Early in Life

In premature babies, PC levels increase via the formation of PC *via* the CDP-choline pathway weeks after birth (156). Since the PEMT is poorly expressed, the rate of formation of AA and DHA molecular species may be restricted, whereas, *in utero*, both choline and PUFAs are supplied indirectly *via* the PEMT pathway of the mother. The supply of both choline and the long-chain PUFAs AA and DHA has, therefore, been advocated (157).

After birth, the plasma level of PC increases during the first 4 months of life (158). At the same time, the proportion of AA in both PC and LPC decreases and linoleate increases (158). Thus, during the intrauterine life during which fatty acids are not used much for energy production, the fatty acid composition of plasma PC is rather similar to that of liver tissue. During suckling, when LA and oleic acid are supplied, elongation-desaturation of LA becomes the major source of tissue ARA and lipoproteins transport more fatty acids for oxidation and energy storage. This is also reflected in the fatty acid composition of PC.

Lyso-PC may also be an important transporter for the supply of choline and PUFAs to the fetus *in utero*. The expression of the Mfsd transporter in the placenta may be of key importance (141). An early study (159) found a remarkably high uptake into the placenta after IV injection of 3H-labeled lyso-PC in pregnant rats.

### Postprandial Metabolism of Ethanolamine

Chyle chylomicrons and nascent VLDL contain a larger proportion of PE than blood lipoproteins (116). After the transfer of PE to HDL, it is rapidly cleared by the action of hepatic lipase (142), which generates highly unsaturated 2-acyl-lyso-PE. The uptake by the liver is high, but lyso-PE also delivers PUFAs and ethanolamine to other tissues (142, 146). However, quantitative studies of the uptake of lyso-PE into the brain and of the role of the mfsd2a transporter in the uptake of lyso-PE into the brain are lacking.

Free ethanolamine in the portal vein is extensively used for hepatic PE synthesis (128). Furthermore, PE synthesis in hepatocytes increases with access to ethanolamine up to a saturation level when ethanolamine-phosphate accumulates (160). In mammalian cells, PE is synthesized by the CDP-ethanolamine pathway and the PS decarboxylation pathway, which operate in the endoplasmic reticulum and mitochondria, respectively (136).

The conclusion is that ethanolamine and PE in milk contribute both to the transport of PUFA and ethanolamine to tissues and to a highly active hepatic PE synthesis, which, in turn, may favor the formation of highly unsaturated PC species *via* PEMT.

### Metabolism of Arachidonic Acid and Long-Chain n-3 Fatty Acid Esters in Chylomicrons

Some AA and DHA are incorporated into the chyle TAGs. The AA and DHA esters of chylomicron TAGs are, in part, resistant to lipoprotein lipase, and are, therefore, transported to the liver with the chylomicron remnants (161, 162). In rats, chylomicron AA contributes significantly to bile PC-AA and mucosal AA pools *via* bile secretion (118).

## Post-Prandial Effects of Milk Polar Lipids

The potential health effects of milk polar lipids have recently been reviewed (4, 163). Here, we commented on the post-prandial effects that milk polar lipids may have on post-prandial PL and TAG metabolism. Animal studies have shown that feeding PC decreases the size of chylomicrons and increases the PC/TAG ratio of the post-prandial lipoproteins [for ref see Nilsson and Duan (6)]. This, in turn, may be linked to a raise in HDL-PC and the size of HDL particles [for see Anto and Warykas (4)].

The numerous factors that influence the post-prandial response to lipids in a meal have been summarized (164). With a Western diet rich in butter fat, the post-prandial TAG-rich lipoprotein particles were more plentiful and smaller than with a Mediterranean-type diet (165). Ingestion of 42–50-g butter resulted in lower post-prandial TAG than the equivalent amount of olive oil in men (166). The lower increase in plasma TAG after intake of butter can be related to the course of digestion and the fatty acid composition with high-content saturated and medium- and short-chain fatty acids (166). Studies of different dairy products have shown that cream cheese induced a larger increase in post-prandial TAG than butter and cheddar cheese, which was suggested to be linked to the disintegration rate of the product in the gut (167).

When comparing the post-prandial effects of soy and milk PL on lipid metabolism in mice, emulsification with PL resulted in a faster increase in plasma TAG but also in a faster plasma clearance and lower TAG levels after 4 h. The findings were correlated with a faster duodenal lipolysis of the milk PL-emulsified lipids (168). Interestingly in an 8-week study on obese patients who included either 40-g milk fat as butter oil or whip cream in their diets, the whip cream group did not exhibit the negative effects on lipid parameters as much as the butter oil group (169). Thus, the question of whether a faster course of absorption favors the partitioning of fatty acids to hepatic oxidation is of great interest.

A lipidomic study analyzed post-prandial plasma lipids after intake of a dairy meal (cheddar cheese, butter, and creamy whole milk) compared to a soy meal with vegetable oil. Both meals contained 54-g fat (170). No difference was seen in post-prandial TAG or cholesterol levels. Although one would expect an increased production of chylomicron PC post-prandially, PC levels were only modestly increased after the dairy meal and unchanged after the soy meal. Changes in distinct molecular species reflected the presence of odd-chain fatty acids in milk and the increased level of LA in the soy lipids. Lyso-PC levels were unchanged after the dairy meal and decreased after the soy meal. Earlier studies showed that, when a mixed meal with 0–50-g fat was given, post-prandial total PL levels decreased significantly after a fat-free meal and increased slightly only with the higher fat doses (171). An increased production of chylomicron and VLDL PC after a meal may thus be balanced by increased post-prandial elimination of PLs. Furthermore, PE and lyso-PE increased similarly after both meals, reflecting the secretion of PE in nascent chylomicrons and VLDL (170). Several SM species increased after the dairy meal and decreased after the soy meal. Although this might reflect a transfer of intact milk SM into blood, it may also result from the high content of palmitic acid in the milk fat and the effects of saturated fatty acids on lipoprotein formation.

Differences in minor PL fractions were observed, which may be potentially relevant, e.g., there was a 34% increase of lyso-PI after the dairy meal but only a 10% increase after the soy meal, which may have implications for fat deposition (172). Chylomicron-PI is a small fraction in chyle but is rich in ARA and is metabolized slower than PE, mainly by hepatic uptake and possibly hepatic lipase (173). Furthermore, alkyl-PC, alkenyl-PC, and alkyl-PE were increased after the dairy meal but decreased after the soy meal, which may be of importance since ether lipids and plasmalogens are negatively associated with metabolic disease (174).

In conclusion, polar milk lipids may be linked both to the course of digestion of other milk lipids and to dose-dependent complex changes in the post-prandial PL pattern. Postprandial metabolomics studies with an increasing dose of MFGM polar lipids are so far lacking. Whether milk polar lipids may be linked to other interesting effects of dairy products, such as effects on release of satiety hormones (175) and anti-inflammatory effects (176, 177), is unknown.

## Conclusions and Perspectives

Milk PC and PE are both hydrolyzed by sPLA2 IB in the upper small intestine, but the PC hydrolysis is more extended and PLB is likely to be involved to a larger degree. Both the selective reacylation of lyso-PC and the use of choline and ethanolamine for the production of mucosal and chylomicron PLs are essential for normal lipid absorption and chylomicron production. The later phase of the PC hydrolysis coincides with SM hydrolysis by alk-SMase and N-CDase. The hydrolysis of PC and SM in mixed bile salt/PL/cholesterol micelles favors cholesterol absorption, which is specifically inhibited by the presence of SM. The dose of MFGM polar lipids required to demonstrate a significant effect in humans is relatively large.

In neonates, the MFGM provides physical properties to the milk fat globule that enhances TAG digestion. The proportion of polar lipids in milk fat globules is much lower than in the chyle lipoproteins. Reacylation of lyso-PC from milk PC is also a contributing but not a major source of PC for chylomicron secretion; mucosal PL synthesis and reacylation of lyso-PC from bile PC must also contribute. Although PLRP2, alk-SMase, and N-CDase are expressed at birth, the course of polar milk lipid digestion in neonates is poorly characterized.

The access of the gut to milk polar lipids and water-soluble choline and ethanolamine favors mucosal and hepatic PC and PE generation. The ARA from milk PE and TAG is selectively retained in mucosal PC, PE, and PI, and is selectively transported with chylomicron PC, PE, and chylomicron remnants. This favors the uptake of ARA by the liver. Furthermore, LCAT probably generates both saturated and unsaturated lyso-PC species. The metabolism of chylomicron PLs and TAGs after feeding milk fat thus contributes nitrogen bases and PUFAs to both the high PL turnover in the liver and to the lyso-PC formation that transports both fatty acids and nitrogen bases to extrahepatic tissues, including the brain. Recent studies have exemplified that analysis of time courses using stable isotopes (156, 178) will clarify the nutritional, diurnal, and developmental regulations of these pathways in the near future.

Other important areas for future studies are the possible links between SM supply and digestion and SIP paracrine signaling between the supply of choline PLs and epithelial acetyl-choline formation. The links between the supply of PE and SM and the trophic and antioxidative effects of ethanolamine are also of interest.

Although interactions with the gut microbiome are important, it is also worth emphasizing that the pancreatic and mucosal enzymes together optimize the digestion of MFGM PLs in a way that normally restricts bacterial access to the ethanolamine and choline parts.

## Author Contributions

All authors listed have made a substantial, direct and intellectual contribution to the work, and approved it for publication.

## Conflict of Interest

The authors declare that the research was conducted in the absence of any commercial or financial relationships that could be construed as a potential conflict of interest.

## Publisher's Note

All claims expressed in this article are solely those of the authors and do not necessarily represent those of their affiliated organizations, or those of the publisher, the editors and the reviewers. Any product that may be evaluated in this article, or claim that may be made by its manufacturer, is not guaranteed or endorsed by the publisher.

## Abbreviations

Alk-SMase: Alkaline sphingomyelinase
ARA: Arachidonic acid
BSSL: Bile salt-stimulated lipase
DHA: Docosahexaenoic acid
LA: Linoleic acid
N-CDase: Neutral ceramidase
PC: Phosphatidylcholine
PE: Phosphatidylethanolamine
PL: Phospholipid
SM: Sphingomyelin.
